# The Blood–Brain Barrier in Health and Disease

**DOI:** 10.3390/ijms24119261

**Published:** 2023-05-25

**Authors:** Sweilem B. Al Rihani, Yazan S. Batarseh, Amal Kaddoumi

**Affiliations:** 1 Tabula Rasa HealthCare, Precision Pharmacotherapy Research and Development Institute, Orlando, FL 32827, USA; srihani@trhc.com; 2 Department of Pharmacology and Biomedical Sciences, Faculty of Pharmacy and Medical Sciences, University of Petra, Amman 1196, Jordan; yazan.s.batarseh@gmail.com; 3 Department of Drug Discovery and Development, Harrison College of Pharmacy, Auburn University, 720 S Donahue Drive, Auburn, AL 36849, USA

The blood–brain barrier (BBB) is a complex network of tightly regulated cells and transport proteins that separate the circulating blood from the brain tissue. This barrier plays a critical role in maintaining a stable and healthy environment for neurons to function by preventing the entry of harmful substances, such as toxins and pathogens, into the brain and removing brain waste products [1]. Furthermore, the BBB is a vital part of the intricate neurovascular system known as the neurovascular unit (NVU), connecting neurons with other essential cells to facilitate their function and structure integrity. NVU consists of endothelial cells that comprise the blood vasculature of the brain, pericytes that surround and maintain the integrity of these vessels, and astrocytes that provide structural and metabolic support to neurons [1,2]. Any disturbance in the balance of the BBB and NVU may lead to pathological alterations in brain function that could be linked to multiple presentations of neurodegeneration and deteriorating brain function. Moreover, the BBB can also pose challenges to the delivery of therapeutic agents for the treatment of various neurological disorders, including biological treatments that involve monoclonal antibody utilization [3]. This Special Issue opens the platform for understanding the BBB and its role in health and disease, which is crucial for preventing and developing effective treatments for neurological disorders.

This Special Issue entitled “The Blood–Brain Barrier in Health and Disease” of the *International Journal of Molecular Sciences* includes two original research articles and four reviews providing new insights into the crucial role of the BBB in brain function and dysfunction.

A critical review by Erickson et al. [4] discusses the scientific evidence of the interaction between the severe acute respiratory syndrome coronavirus 2 (SARS-CoV-2) and the BBB. The authors summarize the available literature on how SARS-CoV-2 may infect and damage brain cells, particularly endothelial cells, and neurons, and the potential mechanisms of how this virus crosses the BBB and contributes to the neurologic sequelae of COVID-19. The authors suggested a few mechanisms through which the virus crosses the BBB to have access to the brain via retrograde nerve routes similar to the alpha herpes viruses, as an example, or by infecting the immune cells, which enables the virus entry into the brain. Disrupted BBB caused by the SARS-CoV-2 virus has also been suggested as another route for the virus and potentially other toxic molecules, such as inflammatory cytokines, to enter the brain. Moreover, virus-induced inflammation could also lead to BBB disruption, which subsequently leads to a vicious cycle between inflammation and BBB disruption and thus potential cognitive impairment and neurodegeneration. Indeed, additional studies are necessary to confirm the relationship between COVID-19, BBB, and neurodegenerative diseases as well as the mechanisms through which the virus invades the brain.

In another comprehensive review, Al Rihani et al. [5] highlighted the critical role of the BBB in maintaining brain homeostasis and neuroprotection. The authors described the complex structure of the BBB with a particular focus on the interplay between the physiology and pathophysiology of the major superfamilies of transporters expressed at the BBB, including the ATP-binding cassette (ABC) transporters and the solute carrier superfamily (SLC). The authors also reviewed the influence of inflammatory conditions and diseases such as AD, epilepsy, amyotrophic lateral sclerosis (ALS), stroke, and multiple sclerosis on the expression and functionality of important BBB drug transporters, their consequential modulation on drug delivery to the brain, and their impact on drug efficacy, resistance, and toxicity.

Alterations in several ABC transporters highly expressed at the luminal side of the BBB have been extensively studied in AD and other neurodegenerative diseases. Of particular interest is the modulation of the expression and activity of the efflux transporters P-glycoprotein (P-gp, ABCB1), and breast cancer resistant protein (BCRP, ABCG2), the low-density lipoprotein receptor-related protein-1 (LRP-1), and the receptor for advanced glycated end products (RAGE), which regulate molecules’ transportation across the BBB to the blood or vice versa and play essential roles in brain amyloid-β (Aβ) homeostasis. Abdallah et al. [6] specifically investigated the impact of the pharmacological downregulation of BBB-suited P-gp and BCRP on the integrity and function of the BBB in TgSwDI, an AD mouse model. The authors reported that the downregulation of P-gp and BCRP using the investigational compound elacridar exacerbated AD pathology. These findings emphasize the impact of using medications that inhibit those two transporters on the disruption of BBB function, thus increasing the risk for AD. In addition, these results suggest that BBB is a therapeutic target. Several studies have shown that rectifying BBB function by restoring the transporters’ expression and function could be a potential strategy to treat AD and other neurodegenerative diseases characterized by BBB dysfunction [7,8,9].

The review by Salmina et al. [10] focused on the significant role of mitochondria-driven BBB and NVU dysfunction. Functional mitochondria are vital for functional brain cells, including the endothelial cells, pericytes, astrocytes, and neurons, where alterations in the mitochondrial activity could lead to AD and related dementia. To study the mitochondria function and its target as a therapeutic strategy for AD treatment, the authors presented novel BBB and NVU in vitro models such as NVU-on-chip, BBB-on-chip, and 3D NVU/BBB models, as well as brain organoids.

Several studies showed a robust correlation between AD, BBB dysfunction, and cerebrovascular diseases such as ischemic and hemorrhagic stroke. In this regard, Unzeta et al. [11] provided a detailed review of the recent evidence on the interrelationship between stroke and AD, specifically through vascular system dysfunction. The authors suggested a possible pathway involving the transition between these two pathologies through the semicarbazide-sensitive amine oxidase (SSAO), also known as vascular adhesion protein-1 (VAP-1), a multifunctional enzyme that is highly expressed in vessels and involved in inflammation. Hence, the inhibition of the SSAO/VAP-1 activity has been proposed as a potential therapeutic approach to reduce the brain damage induced by AD and stroke.

Furthermore, Moon et al. [12] performed a clinical study to evaluate whether specific regional BBB integrity might differ between males and females and whether aging or cognitive decline could influence these differences in BBB integrity. For this purpose, the authors performed neuroimaging studies using dynamic contrast-enhanced magnetic resonance imaging (DCE-MRI) to evaluate BBB permeability. The study involved 75 subjects with normal cognition or mild cognitive impairment (MCI). The findings revealed that compared with males, females might have better BBB integrity in specific regions, namely the occipital and cingulate cortices. This effect was most prominent during the early stages of neurodegeneration. Interestingly, with aging or increased cognitive decline, this sex-related difference was attenuated except for the occipital cortex region, where the difference remained apparent. In addition, the study findings suggested that in females, cognition is directly affected by alterations in BBB integrity. These results emphasize sex as a significant factor that affects disease risk, cognitive function, and ultimately a response to treatments, an area of research that is limited and requires extensive investigation.

In conclusion, the BBB is an essential component of the central nervous system, which helps to maintain a balanced microenvironment that provides support, structure, and protection to neurons. Its integrity is critical, and any deterioration in its function and structure can lead to the development of various neurodegenerative diseases. The BBB responds dynamically to many events, such as vascular disturbances, oxidative stress-linked free radical release, and neuroinflammatory cytokine production. Furthermore, several neurological disorders are associated with increased BBB permeability. In the presence of diseases affecting the BBB’s function, they can contribute to secondary effects on the NVU, altering their balance and amplifying the original insult. The articles presented in this Special Issue cover various important aspects of the BBB, including its cellular components, the role of pathological stimuli, and several neurological confounding factors that may contribute to BBB impairment.
